# Lytic cell death in chronic lung diseases

**DOI:** 10.1093/ajrcmb/aanag092

**Published:** 2026-04-30

**Authors:** Ioannis Siokas, Morgan Heileman, Lawrence C Fritz, Alexei Degterev

**Affiliations:** Program in Genetics, Molecular and Cell Biology, Tufts University School of Medicine, Boston, MA, United States; Program in Genetics, Molecular and Cell Biology, Tufts University School of Medicine, Boston, MA, United States; Vaayu Therapeutics, Rancho Santa Fe, CA, United States; Program in Genetics, Molecular and Cell Biology, Tufts University School of Medicine, Boston, MA, United States

**Keywords:** cell death, necroptosis, pyroptosis, chronic lung disease, viral infections

## Abstract

Regulated cell death is critical in maintaining tissue homeostasis and immune functions. The most common form of physiologic cell death is the highly precise, regulated, and relatively inflammation-free pathway of apoptosis. In contrast, necrotic or lytic cell death, which involves rapid cell lysis and release of pro-inflammatory mediators, is more often associated with human pathologies. Pyroptosis and necroptosis are 2 regulated forms of lytic cell death and are increasingly recognized as important mediators of tissue damage in many diseases, including lung diseases. The death of lung cells is a key contributor to disease in multiple respiratory disorders. However, current interventions are limited to targeting downstream effects to help relieve symptoms without addressing the root cause driving tissue damage. Therefore, new strategies directed toward preventing tissue damage caused by detrimental cell death are desperately needed. Emerging evidence strongly implicates regulated necrosis in many acute and chronic lung diseases, but the specific form(s) of cell death driving pathology and cell types involved require nuanced evaluation. Here, we summarize current literature suggesting that pyroptosis and necroptosis facilitate lung injury and inflammation in chronic obstructive pulmonary disease and idiopathic pulmonary fibrosis by inducing the death of airway and alveolar epithelial cells and resident immune cells, which leads to alveolar barrier disruption, release of inflammatory mediators, excessive immune cell recruitment, and ultimate lung function decline.

## Graphical Abstract

**Graphical Abstract:**
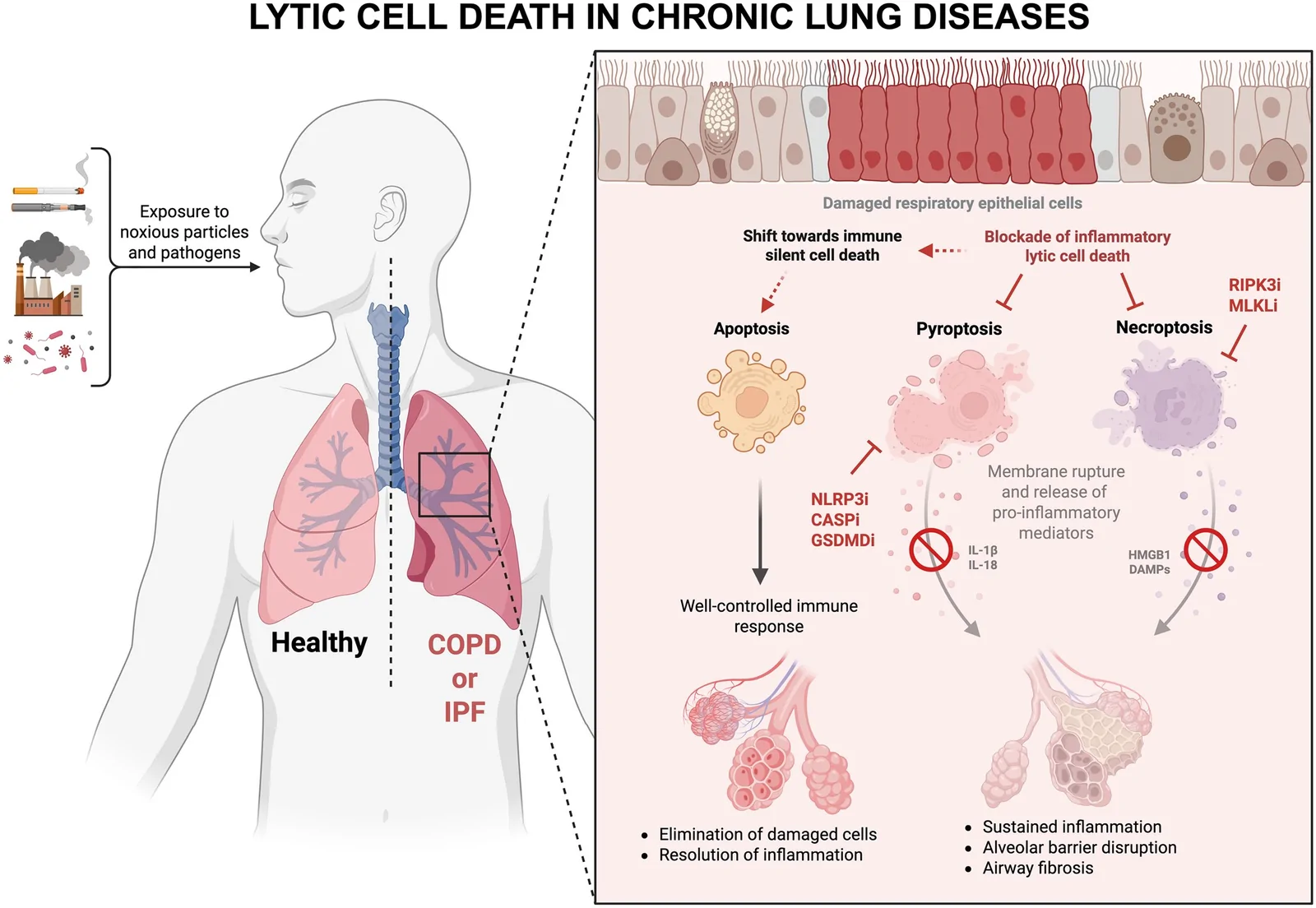

## Overview of regulated lytic cell death mechanisms

Cell death is categorized into apoptotic, regulated lytic (necrotic), and unregulated necrotic forms^1^ (Table 1). Apoptosis is a highly regulated, cysteine protease (caspase)-dependent program triggered by intrinsic or extrinsic cues (Figure 1A). Although sometimes implicated in disease, it is essential for development and tissue homeostasis. Membranes of apoptotic cells and of their remnants (apoptotic bodies) remain largely intact initially and are rapidly cleared by scavenger cells, preventing intracellular contents from being exposed to the immune system and making apoptosis largely noninflammatory. In contrast, lytic cell death, including pyroptosis and necroptosis, features plasma membrane rupture and release of inflammatory damage-associated molecular patterns (DAMPs) and cytokines. In the case of regulated necrosis, these changes are brought about by intrinsic cellular machinery, whereas unregulated necrosis is defined as uncontrollable, catastrophic cell lysis (Table 1).

**Figure 1:**
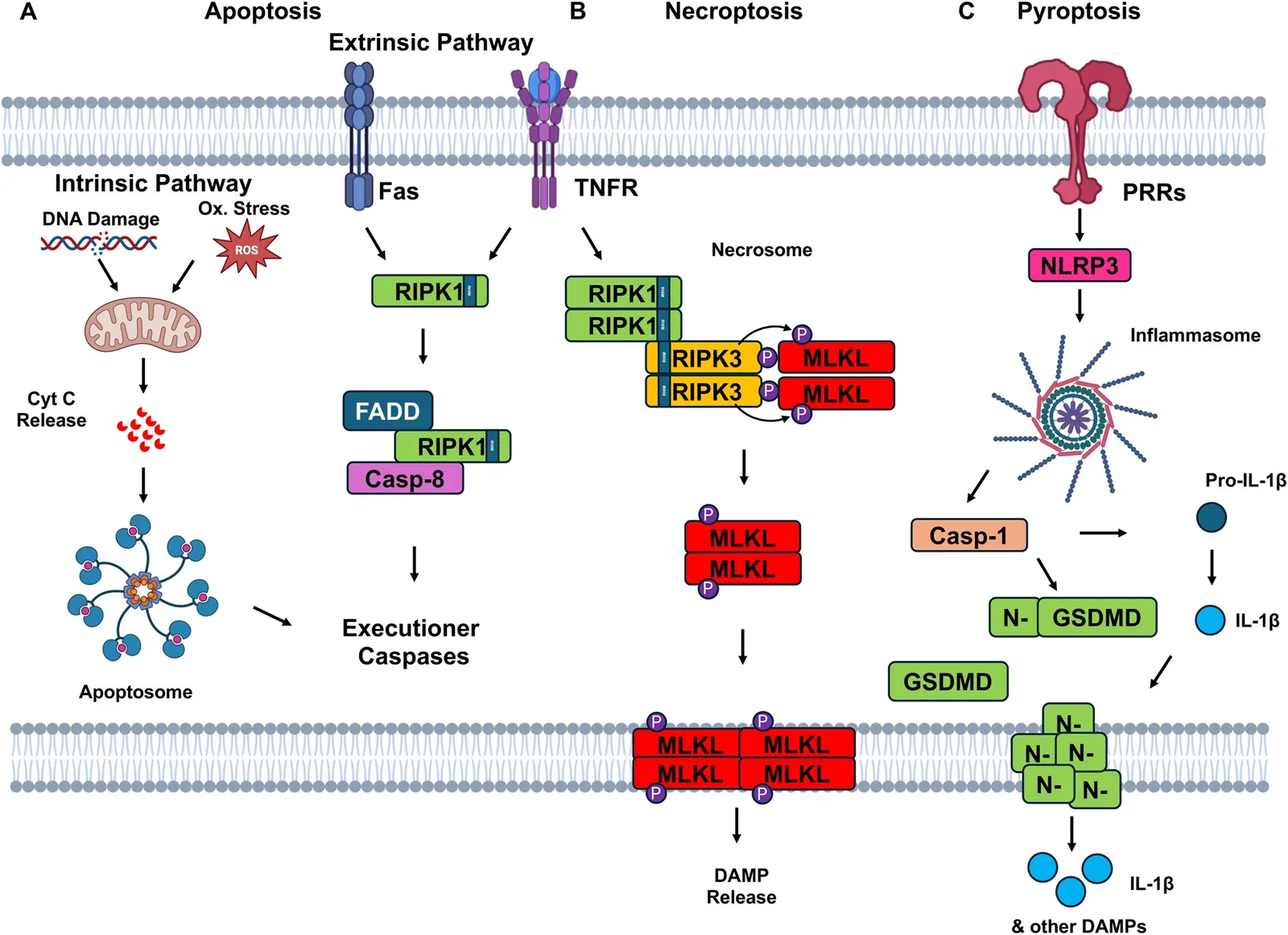
Mechanism of canonical cell death activation. (A) Apoptosis proceeds through 2 main pathways: an intrinsic mitochondrial pathway and an extrinsic death receptor pathway. The intrinsic mitochondrial pathway (left) is initiated by internal stress signals (DNA damage, oxidative stress) and involves mitochondrial outer membrane permeabilization and the release of cytochrome c, which activates the apoptosome and executioner caspases. The extrinsic (death receptor) pathway (right) is triggered by external signals (binding of Fas ligand or TNF-α to their receptors) to activate caspases. (B) Necroptosis can occur downstream of TNF Receptor (TNFR) activation. RIPK1 and RIPK3 assemble the necrosome to initiate MLKL phosphorylation and subsequent oligomerization. MLKL oligomers move to the plasma membrane, where they disrupt its integrity and cause cell lysis and the release of damage-associated molecular patterns (DAMPs). (C) Canonical activation of pyroptosis results from activation of intracellular pattern recognition receptors PRRs, such as NLRP3, which assemble to form the inflammasome. This leads to activation of caspase-1, which initiates pyroptosis by cleaving gasdermin D (GSDMD) and processing of pro-inflammatory cytokines, pro-IL-1β, and pro-IL-18 into their mature forms. The cleaved N-terminal portion of GSDMD assembles into a pore in the plasma membrane releasing cytokines and DAMPs. PRR, pattern recognition receptor.

**Table 1:** Key players in cell death mechanisms.

Cell Death Process	Key Players
**Apoptosis**	**Caspases (-3, -9, -11)** **Bcl-2 family (Bcl-2, Bax, Bak)** **Cytochrome C** **Apaf-1**
**Necroptosis**	**RIPK1, RIPK3 (ph-RIPK1, ph-RIPK3)** **MLKL (ph-MLKL)** **DAMP release**
**Pyroptosis**	**Gasdermins (GSDM-D, E, B, C)** **Inflammasomes (NLRP1, NLRP3, NLRC4, AIM2, ASC, pyrin)** **Caspase-1** **Caspase-4, -5 (human)** **Caspase-11 (mouse)**
**Ferroptosis**	**Glutathione peroxidase 4 (GPX4)** **System Xc- (SLC7A11)** **Iron, lipid peroxides** **ACSL4**
**NETosis**	**Neutrophil elastase** **Myeloperoxidase** **PAD4 (peptidyl arginine deaminase 4)** **Reactive oxygen species (ROS)**
**Unregulated Necrosis**	**Does NOT involve coordinated activation of specialized cellular machinery** **Can be induced by:** **Mechanical damage** **Chemical damage** **ROS** **Loss of ATP or ion imbalance**

Pyroptosis relies on the activation of caspases and gasdermins. In the canonical pyroptotic pathway, pathogen‑associated molecular patterns and DAMPs are recognized by specific inflammasome sensor pattern recognition receptors (PRRs), which, together with appropriate priming and activation signals (“2-hit mechanism”), drive assembly of canonical inflammasomes that induce cleavage and activation of caspase-1. The inflammasomes in pyroptosis use NLRP3, NLRP1, NLRC4, AIM2, and pyrin as their PRRs to detect diverse signals such as cellular stress, cytosolic DNA, bacterial ligands, and toxins.^2^ Gasdermin D (GSDMD) and E are the best-characterized pyroptotic effectors. All gasdermins contain an N-terminal pore-forming domain and a C-terminal inhibitory domain. Activated (cleaved) caspase-1 cleaves and releases the N-terminal gasdermin fragment, which oligomerizes in the plasma membrane to form pores, promoting cell swelling, rupture, and release of inflammatory mediators. Caspase-1 also processes pro-IL-1β and pro-IL-18 into mature cytokines, which are released during pyroptosis (Figure 1C). In noncanonical pyroptosis, cytosolic lipopolysaccharide (LPS) from gram-negative bacteria directly activates caspase-4/5 (and caspase-11 in mice) to cleave gasdermin without inflammasome involvement.^3^ Caspase-8-dependent cleavage of GSDMD has also been described.^4^ Whereas cleaved gasdermins form lethal membrane pores, full membrane rupture and release of all intracellular contents depends on the amphipathic filaments formed by ninjurin-1.^5^

Conversely, necroptosis is caspase-independent and relies on a RIPK1-RIPK3-MLKL signaling cascade. It is activated by innate immune receptors, including TNF-Receptor-1, Toll-like receptors, and nucleic acid sensors.^6^ In the example of TNF-Receptor-1, ligand-induced receptor trimerization promotes formation of a membrane-bound receptor-associated complex (Complex I) including adaptor proteins, E3 ligases, and Receptor-interacting Protein Kinase 1 (RIPK1). Activated RIPK1 moves to the cytosol, forming the death-inducing Complex II with caspase-8 to mediate either caspase-dependent cell death, apoptosis and pyroptosis, or recruit RIPK3 to assemble an oligomeric necroptosis-inducing complex (necrosome).^6^ RIPK3 is also activated directly by ZBP1, a sensor of left-handed, double-stranded nucleic acids.^7^ RIPK3 activation leads to recruitment and phosphorylation of Mixed Lineage Kinase Domain-Like (MLKL). Phosphorylated-MLKL (ph-MLKL) oligomerizes and translocates to the plasma membrane, disrupting membrane integrity and triggering necroptosis with release of DAMPs, cytokines, and chemokines^8^ (Figure 1B). Additional regulated lytic death pathways also exist (Table 1) but will not be discussed in our review.

## Lytic cell death in chronic obstructive pulmonary disease

Chronic obstructive pulmonary disease (COPD) is a chronic lung disease characterized by airway inflammation, mucus overproduction, and destruction of alveolar architecture, resulting in persistent and progressive airflow obstruction. More than 200 million people are affected worldwide, and this number is projected to triple by 2050, representing a major health care burden.^9^ Chronic exposure to cigarette smoke and environmental toxins are the dominant risk factors by promoting structural lung damage and sustained inflammation. However, the specific mechanisms that drive these processes remain incompletely defined, limiting the development of effective lung-protective therapies.

COPD mouse models of cigarette smoke exposure (CSE) provide evidence for NLRP3-mediated pyroptosis and release of IL-1β and IL-18. This creates a pro-inflammatory lung milieu that recapitulates symptoms of human COPD.^10^ Mice deficient in *Nlrp3*, *Caspase-1*, or *Gsdmd*, or treated with the NLRP3 inhibitor MCC950, exhibit reduced pulmonary inflammation following CSE. Additionally, cleaved GSDMD was detected in bronchial airway epithelial (BECs) cells and alveolar macrophages of CSE-exposed mice, and cigarette smoke directly induced pyroptosis of BECs in vitro.^10^^,^^11^ Mechanistically, cigarette smoke damages mitochondria, and mitochondrial reactive oxygen species have been implicated as activators of NLRP3.^12^ These findings suggest that pyroptotic death of BECs and resident macrophages compromises barrier integrity and increases inflammation. Chronic (4–6 months) ozone exposure models also show lung pyroptosis, which is attenuated by the GSDMD inhibitor Disulfiram,^13^ with improvements in leukocyte infiltration, alveolar structural changes, and pulmonary function. However, additional work is needed to define which cell types undergo pyroptosis in chronic ozone exposure and how it shapes long-term inflammation and emphysema.

RNA-sequencing studies further implicate pyroptosis in COPD with observed upregulation of pyroptosis-related genes (*Gsdmd*, *Il-1b*, *Casp-1/4/5*, and the inflammasome adapter *Pycard*) in human COPD lungs compared with healthy and non-COPD smoker lungs.^13^^,^^14^ Expression was primarily enriched in airway epithelial populations and alveolar macrophages, implicating these as the primary sites of pyroptosis in COPD. However, increased expression of inflammasome or pyroptosis genes does not by itself demonstrate gasdermin cleavage or execution of cell death, and direct evidence of pathway activation in patient samples requires further study.

Necroptosis has also been implicated in COPD models. After CSE, mouse lungs showed elevated ph-MLKL that correlated with duration of CSE.^15^ In vitro, although CSE triggered both apoptosis and necroptosis in BECs, only necroptosis triggered inflammation, which was reduced by genetic deletion of *Ripk3* or *Mlkl.*^15^^,^^16^ The release of inflammatory DAMPs such as HMGB1 by necroptotic cells correlates with disease severity in COPD patients.^17^ In vivo, pharmacologic inhibition of necroptosis with GSK’872 lowered ph-MLKL in lung lysates, dampened inflammation, and mitigated airway enlargement after CSE, a defining feature of emphysema. Importantly, activated ph-RIPK3 and ph-MLKL were also elevated in human COPD lungs compared with nonsmokers and smoker controls without COPD, as determined by western blotting and immunohistochemistry.^15^^,^^16^ This evidence points to necroptosis occurring in the lung epithelium in COPD, but further work will be required to identify the specific cell types affected.

Overall, markers of regulated lytic cell death, both pyroptosis and necroptosis, are detected in COPD models and in patient samples. Although definitive evidence of the role of lytic death will require further studies, the existing data suggest that inhibiting pyroptosis and/or necroptosis could be an exciting new treatment option for COPD.

## Lytic cell death in idiopathic pulmonary fibrosis

Idiopathic pulmonary fibrosis (IPF) is a chronic, fatal lung disease characterized by scattered fibrotic lesions throughout the lung. Although its etiology remains unclear, the prevailing model proposes that repeated injury and subsequent death of lung epithelial cells initiates disease by promoting fibroblast activation into myofibroblasts.^18^ Myofibroblasts accumulate and deposit extracellular matrix intended as a wound-healing response. However, excessive deposition progressively impairs lung function. Current antifibrotic drugs slow, but do not stop, lung function decline, and without lung transplantation patients ultimately succumb to respiratory failure.^19^ The disease is further characterized by abnormal epithelial remodeling and alveolar type II epithelial (AT2), cell senescence. AT2 cells are cuboidal cells in the lung alveoli that produce surfactant to reduce surface tension, regulate immune responses, and serve as progenitors for alveolar epithelium repair after injury. Understanding how death of alveolar epithelium provokes extensive fibrotic remodeling remains a critical imperative.

Lytic cell death provides a promising link between epithelial cell injury and fibroblast activation in IPF. DAMPs from dying epithelial cells can induce proinflammatory and pro-fibrotic remodeling in fibroblasts.^18^ HMGB1, a DAMP released during necroptosis, is elevated in bleomycin (BLM)-induced pulmonary fibrosis in mice, where it promoted IL-1β and TGF-β1 signaling in fibroblasts, enhancing extracellular matrix production, and HMGB1 neutralization reduced fibrosis.^20^

Several gene expression studies identified upregulation of pyroptosis- and necroptosis-related genes in IPF patient lungs and animal models.^21^ Increased pulmonary IL-1β and caspase-1 during early inflammatory phases in BLM-treated mice suggested potential pyroptosis involvement, and single-cell transcriptomic analyses of human IPF lungs revealed increased gasdermin expression in epithelial and stromal cells.^14^ However, upregulation of pyroptotic machinery does not necessarily indicate actual cell death by pyroptosis. The best functional evidence comes from the Toll-Like-Receptor-9 (TLR9)-NLRP3 axis in BLM-treated mice. *Tlr9* knockdown decreased pulmonary fibrosis, and cleaved GSDMD and caspase-1.^22^ Conversely, NLRP3 activation by Nigericin counteracted this protection. Though the cell type in vivo is unknown, TLR9-NLRP3-pyroptosis by BLM was identified in a human alveolar epithelial cell line.

There is more compelling evidence that necroptosis contributes to IPF. Total RIPK3 and ph-MLKL are elevated in IPF patient lungs relative to healthy controls.^23^ In BLM models, necroptosis activation occurred in a RIPK3-dependent manner and coincided with increased HMGB1 and IL-1β. Inhibition of RIPK1, an upstream activator of RIPK3, with Nec-1, or genetic deletion of *Ripk3*, reduced lung inflammation and fibrosis.^23^ A familial IPF-causing mutation was also identified in the lung surfactant protein SFTPA1. *Sftpa1*-mutant mice developed spontaneous IPF-like pathology with heightened necroptosis in AT2 cells.^24^ JNK (a stress sensor upstream of necroptosis) inhibition decreased RIPK3 expression and ameliorated lung fibrosis in mutant mice, whereas RIPK3 overexpression abrogated this effect.

Secondary insults during IPF that induce necroptosis may further exacerbate disease. Intratracheal LPS in BLM-treated mice worsened tissue destruction and fibrosis, with increased ph-MLKL but no cleaved-caspase 3, specifically implicating necroptosis.^25^ Infiltrating CD68+ macrophages were identified as the primary ph-MLKL-positive population, though additional cell types could also be involved. In *Sftpa1*-mutant mice, influenza A virus (IAV) accelerated fibrosis, and deletion of *Ripk3* or *Mlkl* protected against IAV-exacerbated fibrosis and mortality.^24^ Selective RIPK3 inhibition by UH15-38 during IAV infection improved survival and reduced fibrotic remodeling.^26^ Therefore, inhibiting necroptosis in IPF may provide a promising therapeutic strategy complementary with current antifibrotic therapies.

### Therapeutic implications and current targeting strategies

Although the role of epithelial cell death in IPF and COPD is well appreciated, little progress has been made to develop efficient lung-protective treatments for human use (Figure 2). Reasons include limited cell death data in human samples and ex vivo models, lack of clarity on which forms of cell death are critical to target, a need for rational patient stratification methods, and a limited repertoire of proven clinical-stage assets. Nevertheless, inhibition of lytic cell death is increasingly seen as an exciting therapeutic strategy, and multiple pathway targets are under pharmaceutical development, though no clinical testing has yet been done in respiratory diseases. Necroptosis has mainly been targeted through RIPK1 inhibition. First-in-human inhibitors such as GSK2982772 and DNL104 were well tolerated but have not shown clear efficacy in trials for IBD, psoriasis, amyotrophic lateral sclerosis, or multiple sclerosis, motivating alternative approaches such as targeting RIPK3.^27-29^ Although RIPK3 has proven difficult to target from a medicinal chemistry perspective, significant progress has recently been made.^26^ For pyroptosis, NLRP3 is a leading target with several inhibitors in clinical development due to its wide range of stimuli and association with multiple diseases.^30^ Other promising strategies include blocking gasdermins or caspase-1, although caspase-1 inhibitors have been limited by specificity and toxicity concerns.

**Figure 2:**
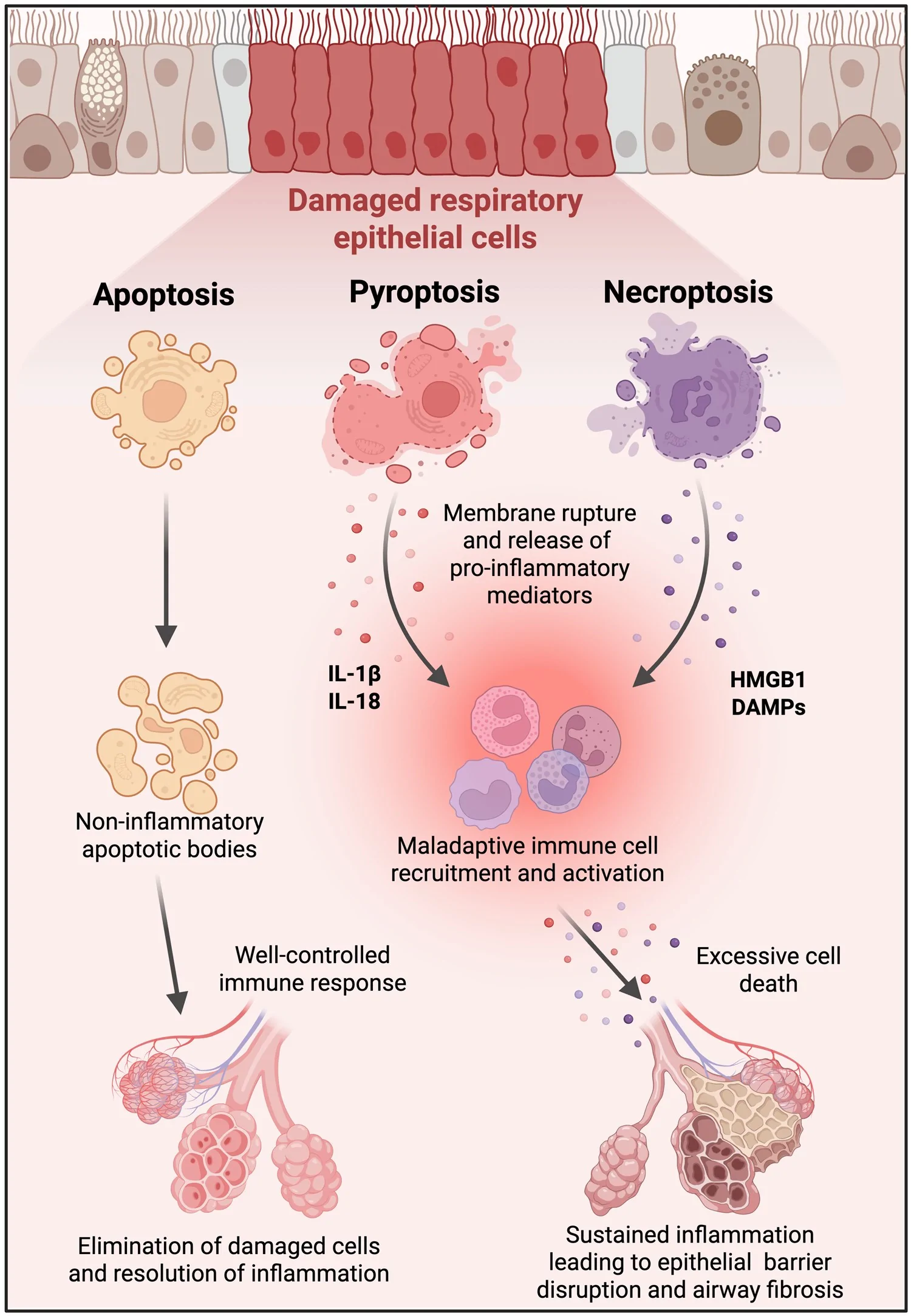
Schematic model illustrating pathways of cell death in lung disease. Damage to the respiratory epithelium activates cell death in airway (pictured) and alveolar epithelial tissue. Cell death via apoptosis leads to a well-controlled immune response and the noninflammatory clearance of damaged or infected cells. However, lytic forms of cell death, such as pyroptosis or necroptosis, may propagate further inflammation in recruited immune cells, leading to excessive tissue damage and loss of lung function, which may contribute to the development of chronic pulmonary diseases. Image created using BioRender.

### Treating chronic lung diseases with cell death inhibitors—how do we get there?

Preclinical lung disease models indicate that inhibiting lytic cell death could be therapeutically valuable, but key challenges remain. Cell death pathways are highly redundant, with distinct modes occurring simultaneously in different lung cell populations, and pyroptosis and necroptosis may be coactivated within the same disease context. Evidence remains limited regarding which lung cell lineages undergo lytic death and which pathway(s) drive pathology, making it essential to define the dominant death programs. This is complicated by shared upstream signaling. RIPK1-containing complexes enable cells to switch programs when 1 pathway is blocked. Importantly, such switching may be advantageous. During IAV infection, suppressing necroptosis while permitting apoptosis improves host outcomes,^26^ suggesting that selective blockade of lytic death could be effective even when other death programs continue. Additionally, the relative contribution of individual pathways likely differ between animal models and humans, and across patient populations. Human-relevant systems capturing disease heterogeneity, such as precision-cut lung slices and 3-dimensional organoids, may help bridge translational gaps. Finally, although “master regulator” targets (caspases or RIPK1) are appealing, they have not delivered clear clinical efficacy. Targeting the more conserved terminal mechanisms, such as MLKL or specific gasdermins may offer improved translatability. Overall, a concentrated effort addressing these gaps is essential to accelerate lung cell-protective therapies.

## Supplementary Material

aanag092_Supplementary_Data
